# Supplementary material for: Tuning the Cytotoxicity of Bis-Phosphino-Amines Ruthenium(II) Para-Cymene Complexes for Clinical Development in Breast Cancer
Source: Pharmaceutics. 2021 Sep 26;13(10):1559. doi: 10.3390/pharmaceutics13101559 (PMC8539368; doi:10.3390/pharmaceutics13101559)
Supplement: Supplementary file 1 [file pharmaceutics-13-01559-s001.zip › pharmaceutics-1319708-SI.pdf]

Supplementary Materials: Tuning the Cytotoxicity of bis-phosphino-amines Ruthenium(II) para-cymene complexes for clinical development in Breast Cancer

Elena Domínguez-Jurado, Francisco J. Cimas, José Antonio Castro-Osma, Alberto Juan, Agustín Lara-Sánchez, Antonio Rodríguez-Diéguez, Alexandr Shafir, Alberto Ocaña and Carlos Alonso-Moreno

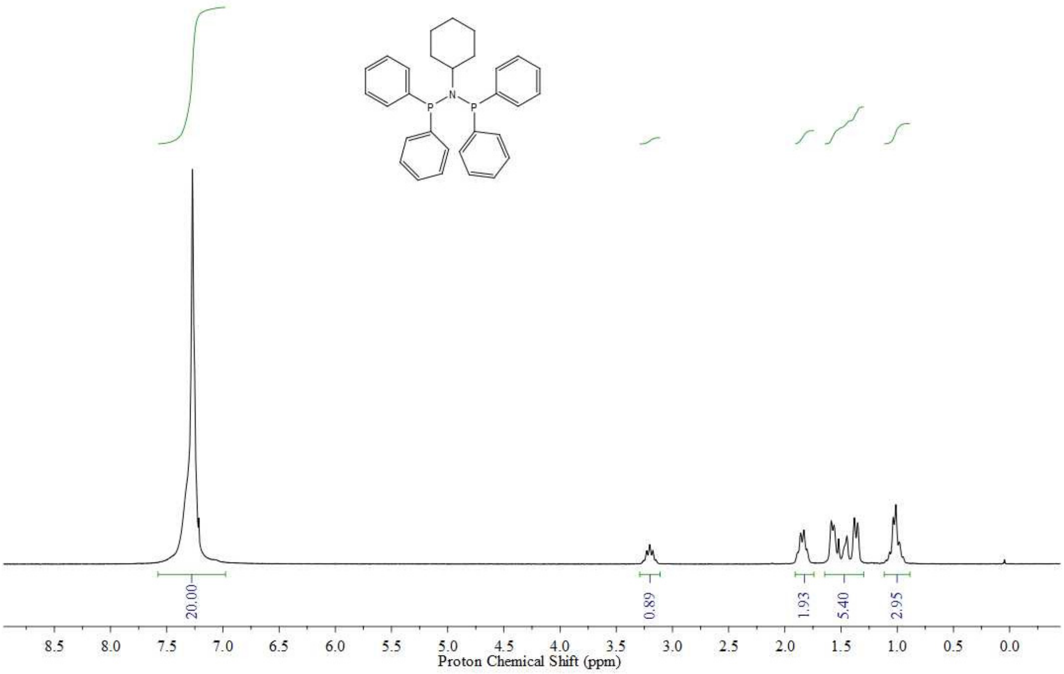

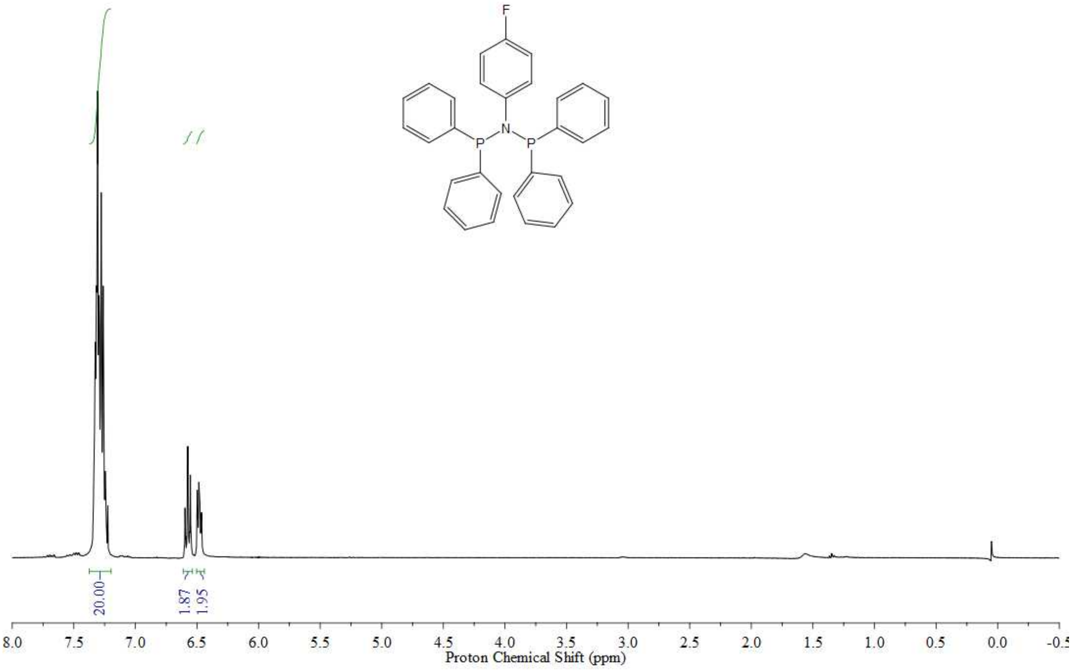

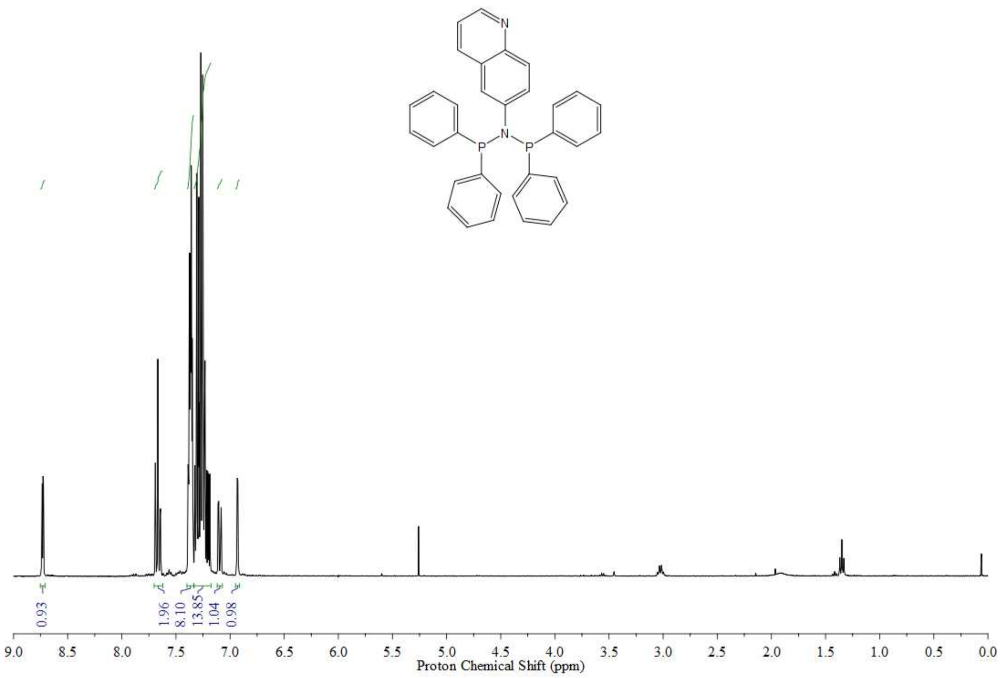

Figure S1. ^{1}H NMR (400 MHz) spectrum of Ligands L3 ($\{Ph_2P\}_2N\{C_6H_{11}\}$), L6 ($\{Ph_2P\}_2N\{C_6H_4F\}$) and L9 ($\{Ph_2P\}_2N\{C_9H_6N\}$) in $CDCl_3$.

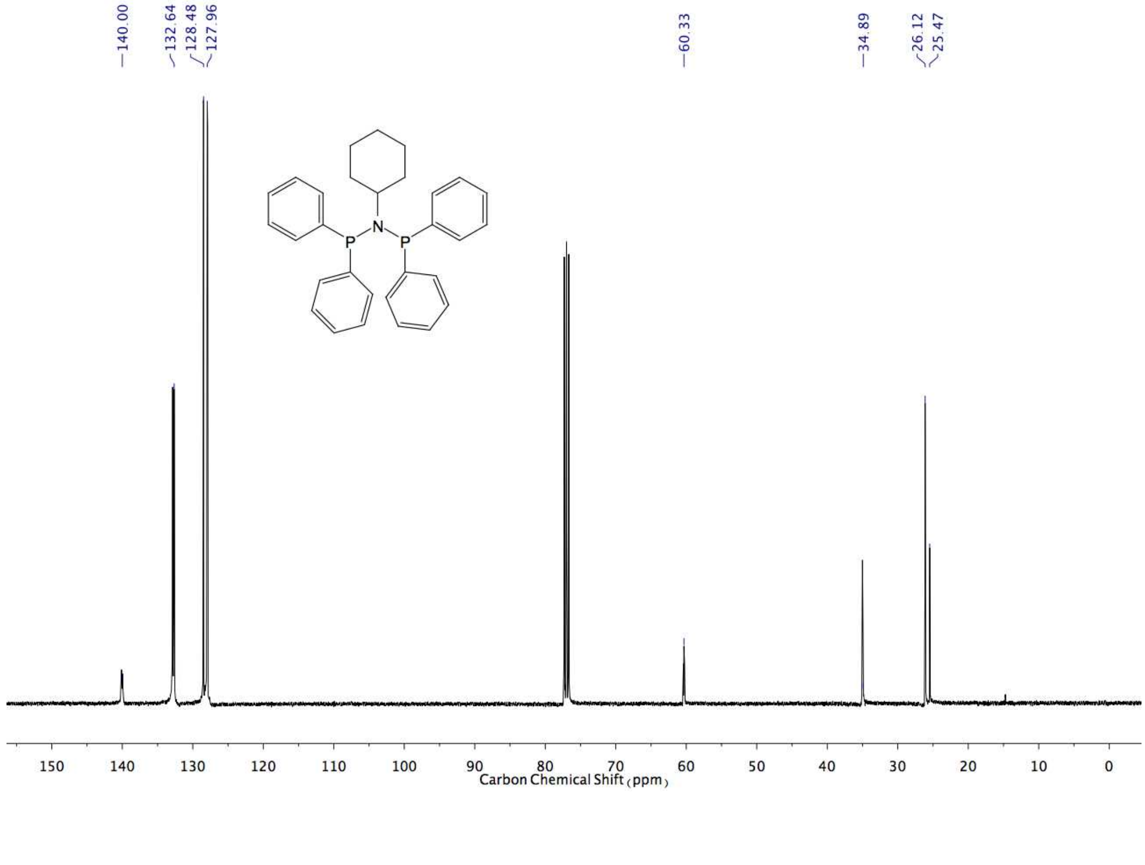

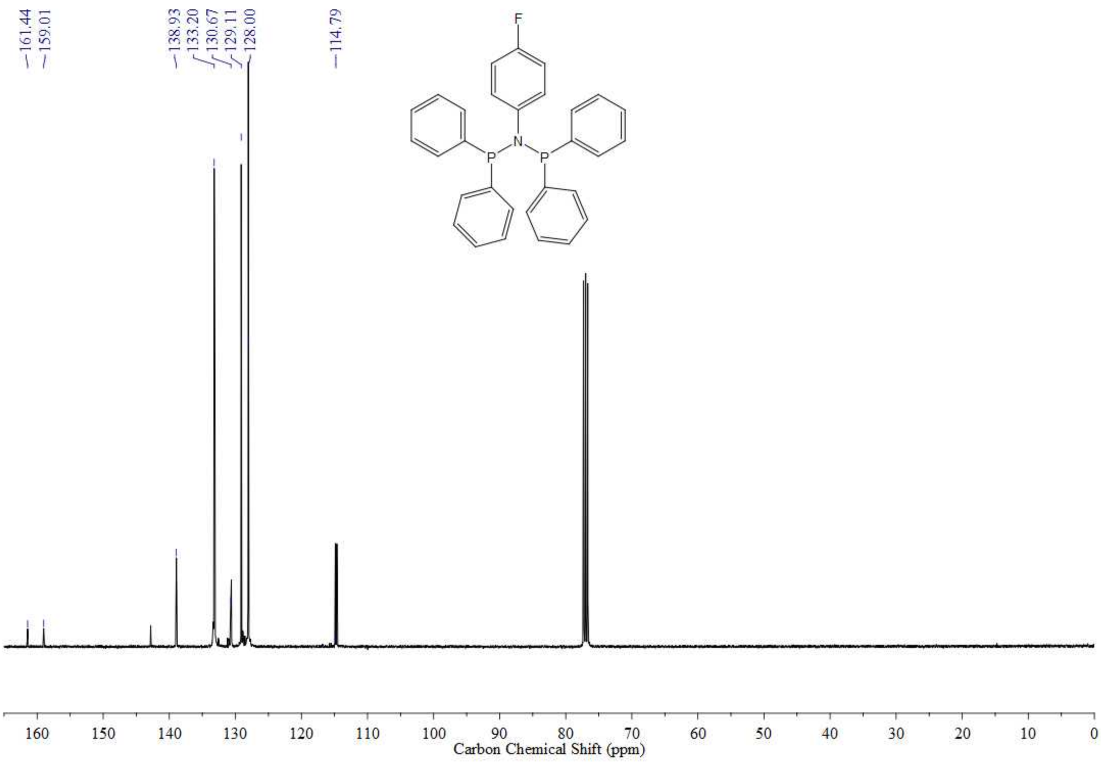

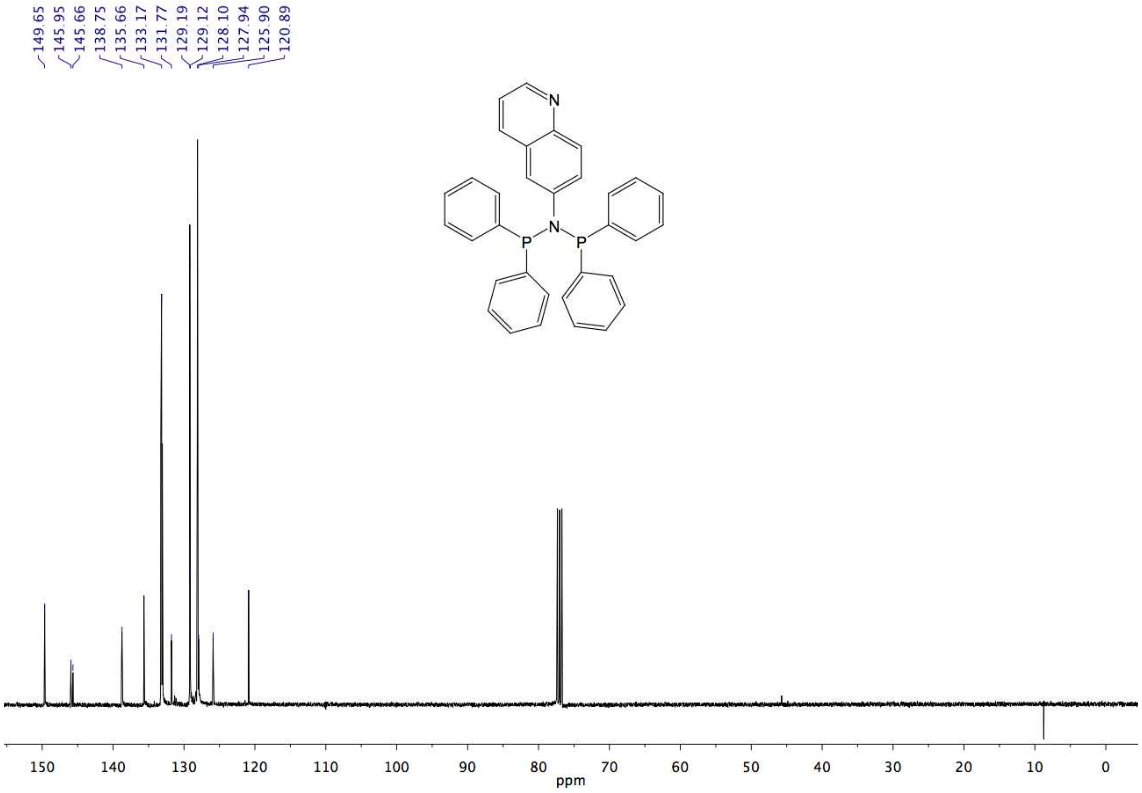

Figure S2. ^{13}C NMR (100 MHz) spectrum of Ligands L3 ($\{Ph_2P\}_2N\{C_6H_{11}\}$), L6 ($\{Ph_2P\}_2N\{C_6H_4F\}$) and L9 ($\{Ph_2P\}_2N\{C_9H_6N\}$) in $CDCl_3$.

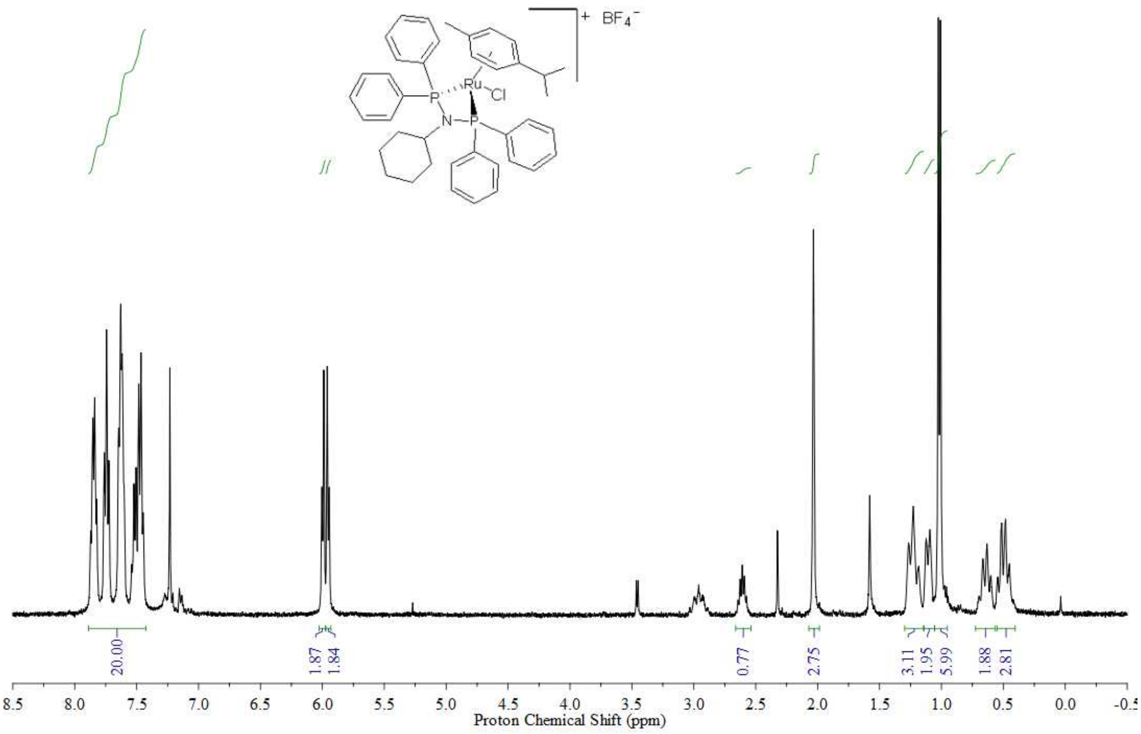
BF4−
Ru
Cl
P
N
20.00
1.87
1.84
0.77
2.75
3.11
1.95
5.99
1.88
2.81
8.5 8.0 7.5 7.0 6.5 6.0 5.5 5.0 4.5 4.0 3.5 3.0 2.5 2.0 1.5 1.0 0.5 0.0 -0.5
Proton Chemical Shift (ppm)

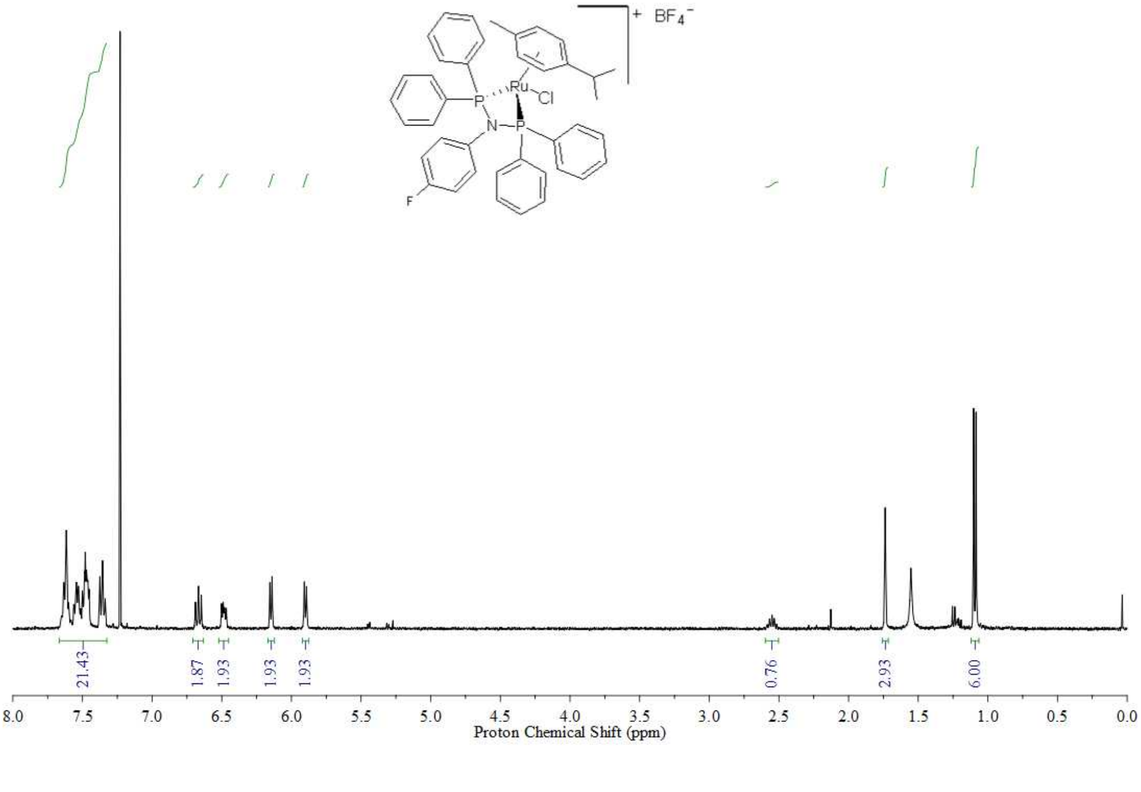
BF4−
Ru
Cl
P
N
F
21.43
1.87
1.93
1.93
1.93
0.76
2.93
6.00
8.0 7.5 7.0 6.5 6.0 5.5 5.0 4.5 4.0 3.5 3.0 2.5 2.0 1.5 1.0 0.5 0.0
Proton Chemical Shift (ppm)

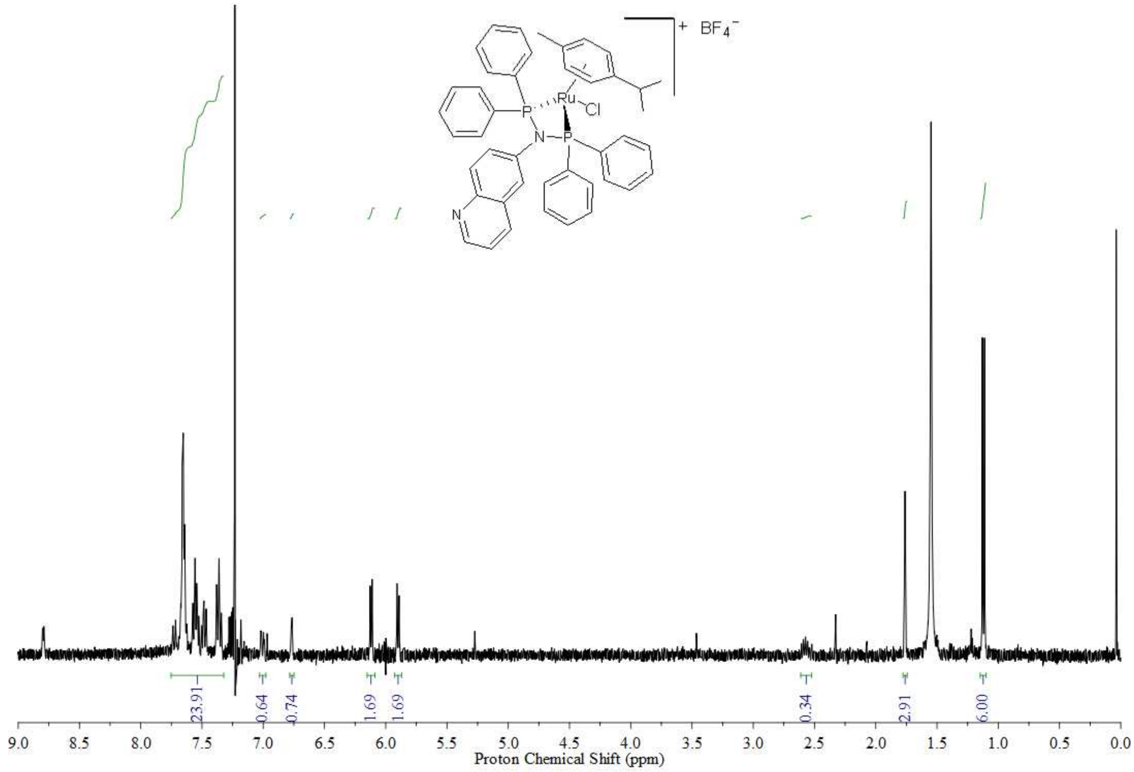

Figure S3. ^{1}H NMR (400 MHz) spectrum of Complexes Ru3 ([RuCl(p-cym)({Ph_2P}$_2$N{C_6H_{11}})][BF_4]), Ru6 ([RuCl(p-cym)({Ph_2P}$_2$N{C_6H_4F})][BF_4]) and Ru9 ([RuCl(p-cym)({Ph_2P}$_2$N{C_9H_6N})][BF_4]) in $CDCl_3$.

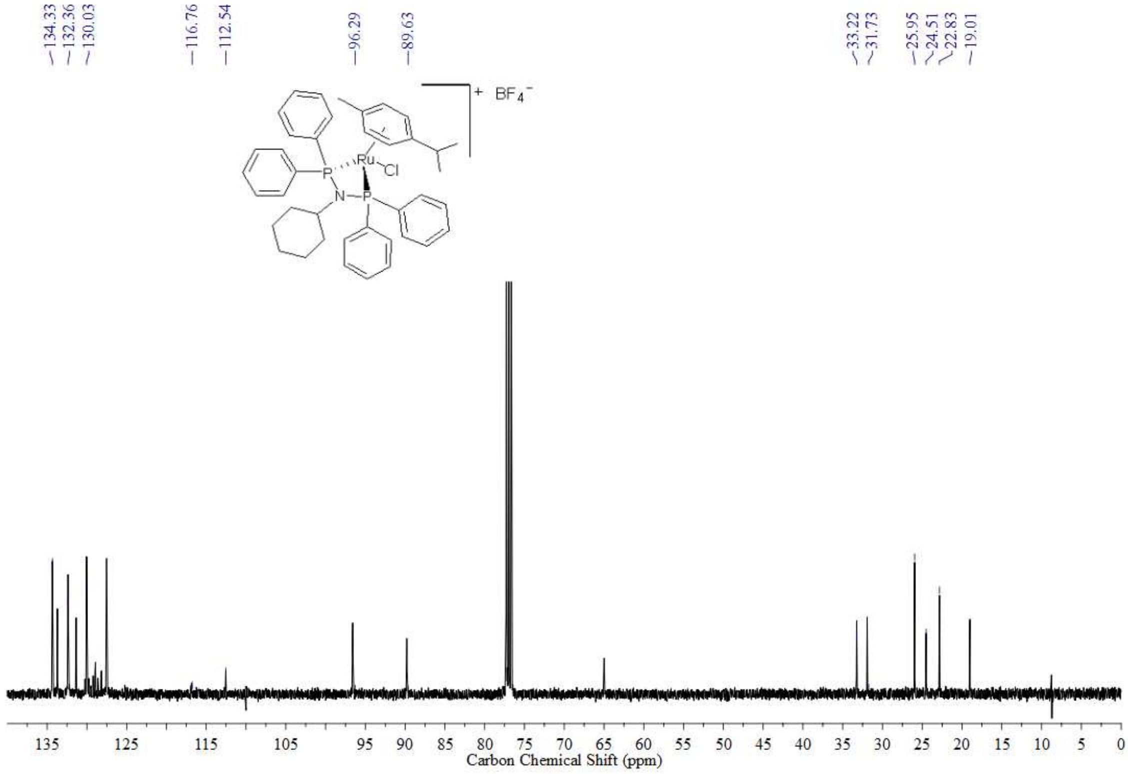

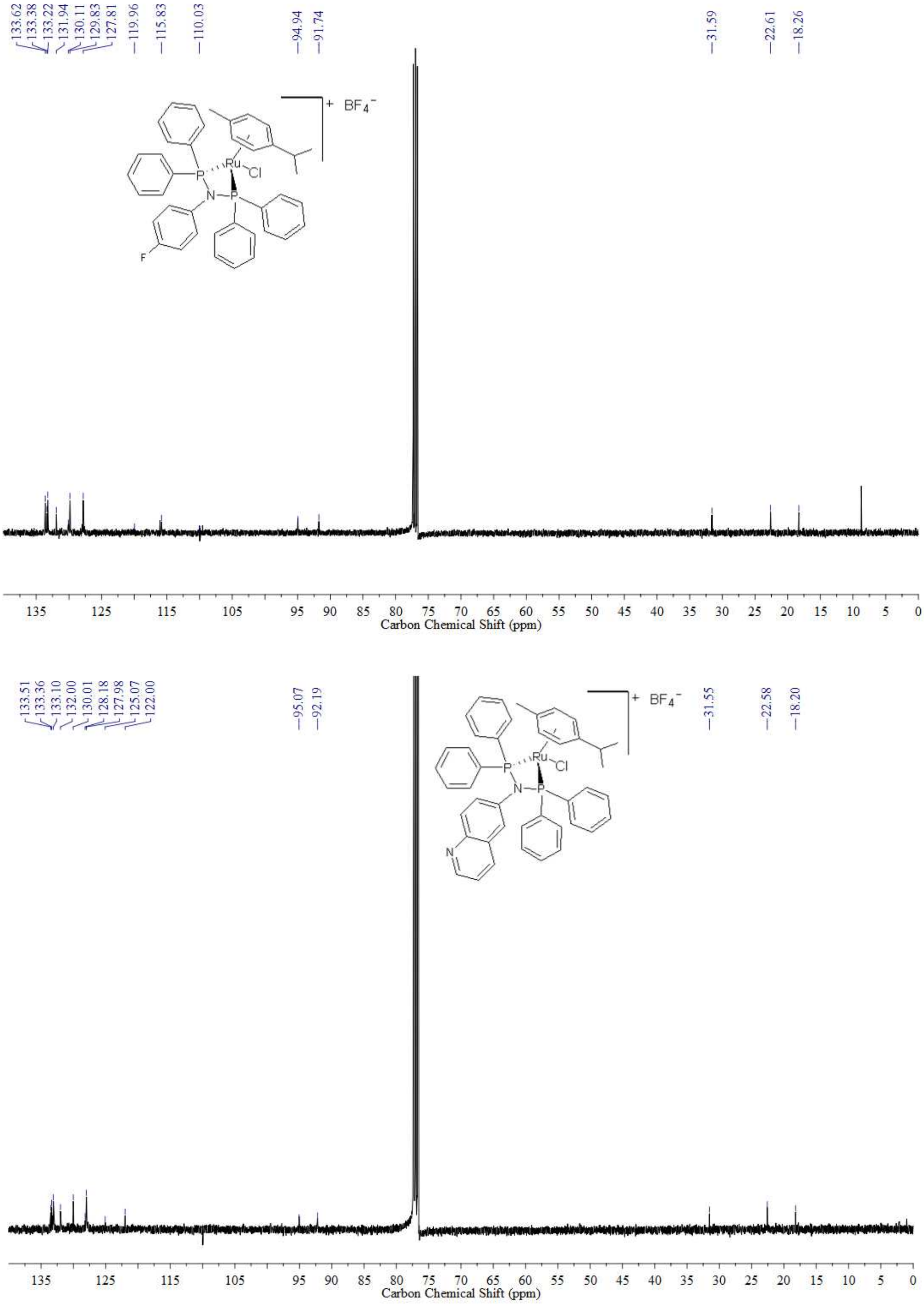

Figure S4. ^{13}C NMR (100 MHz) spectrum of Complexes Ru3 ([RuCl(p-cym)({Ph_2P}$_2$N{C_6H_{11}})][BF_4]), Ru6 ([RuCl(p-cym)({Ph_2P}$_2$N{C_6H_4F})][BF_4]) and Ru9 ([RuCl(p-cym)({Ph_2P}$_2$N{C_9H_6N})][BF_4]) in $CDCl_3$.

Equipo MAXIS II

**Muestra**	Ru1 disuelta 1mg/mL en DCM. Dilucion 5:1000 con MeOH
**Nombre registro**	D:\Data\2021\2021_09 SEPTIEMBRE\MAX4155_5_01_3309.d
**Metodo**	ESI Positive fia esi + 50-2000.m
**Ref archivo**	MAX4155

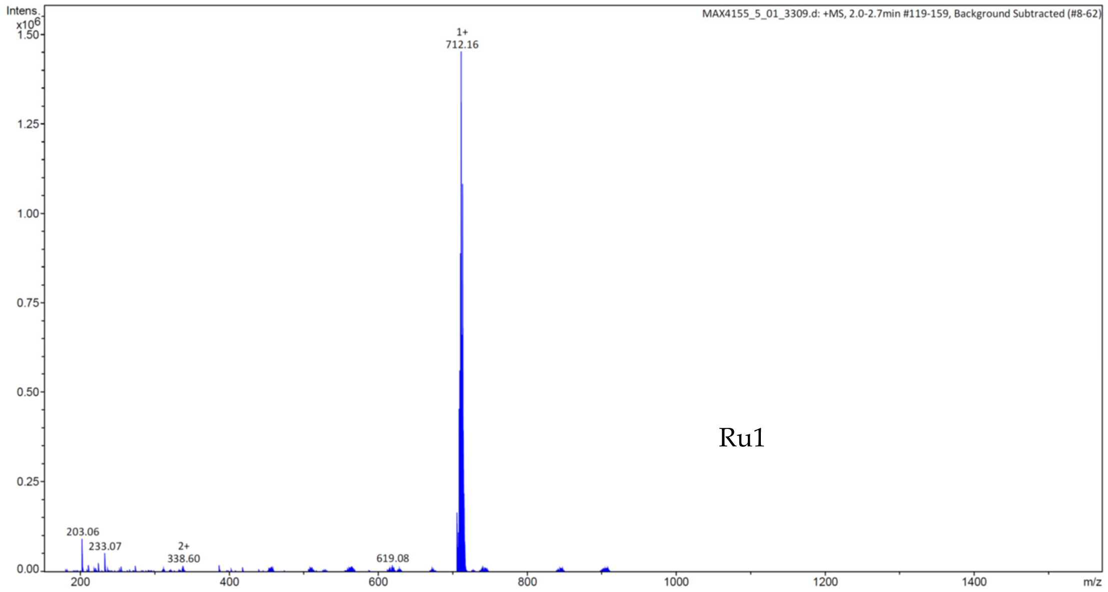

**Muestra**	Ru2 disuelta 1mg/mL en DCM. Dilucion 5:1000 con MeOH
**Nombre registro**	D:\Data\2021\2021_09 SEPTIEMBRE\MAX4156_6_01_3310.d
**Metodo**	ESI Positive fia esi + 50-2000.m
**Ref archivo**	MAX4156

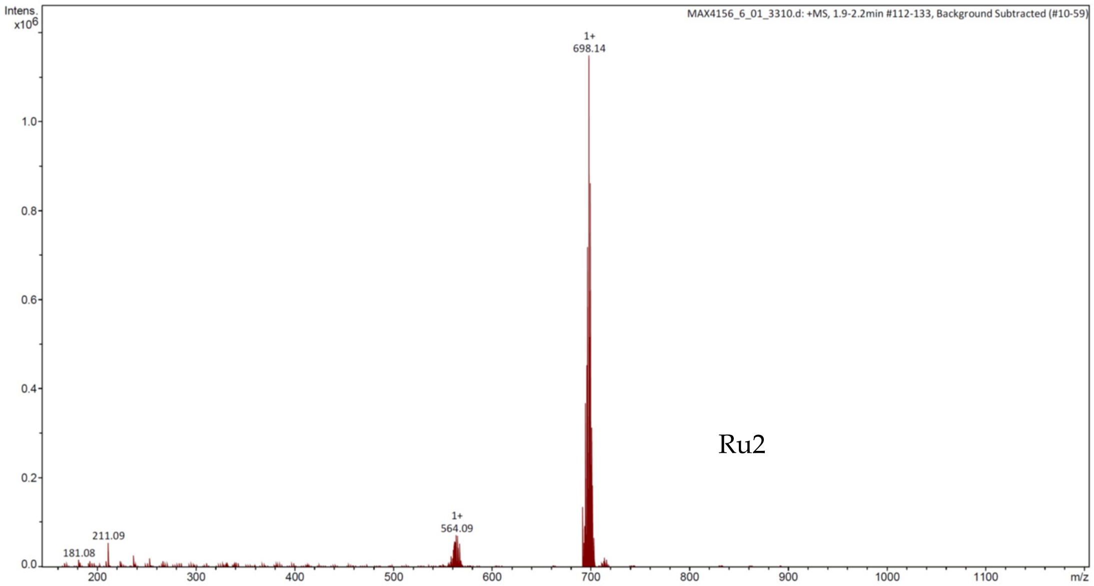

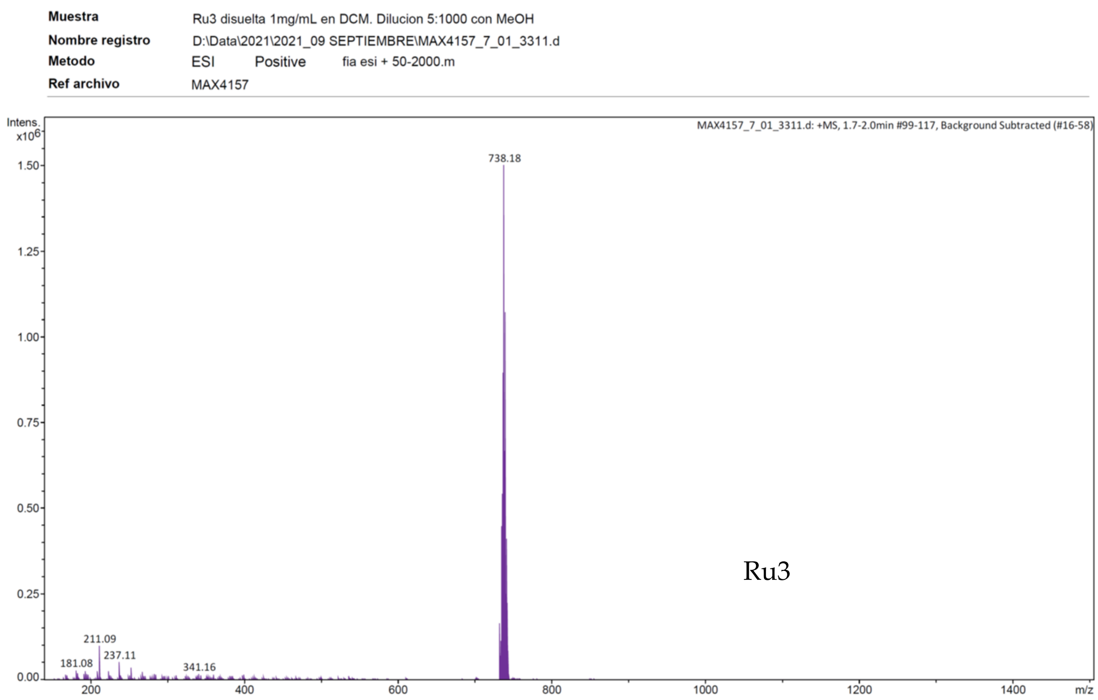

Figure S5. MS(ESI) spectra for Ru1 [RuCl(p-cym)({Ph_2P}$_2$N{C_4H_9})][BF_4] (Ru1), Ru2 [RuCl(p-cym)({Ph_2P}$_2$N{C_3H_7})][BF_4], and Ru3 [RuCl(p-cym)({Ph_2P}$_2$N{C_6H_{11}})][BF_4].

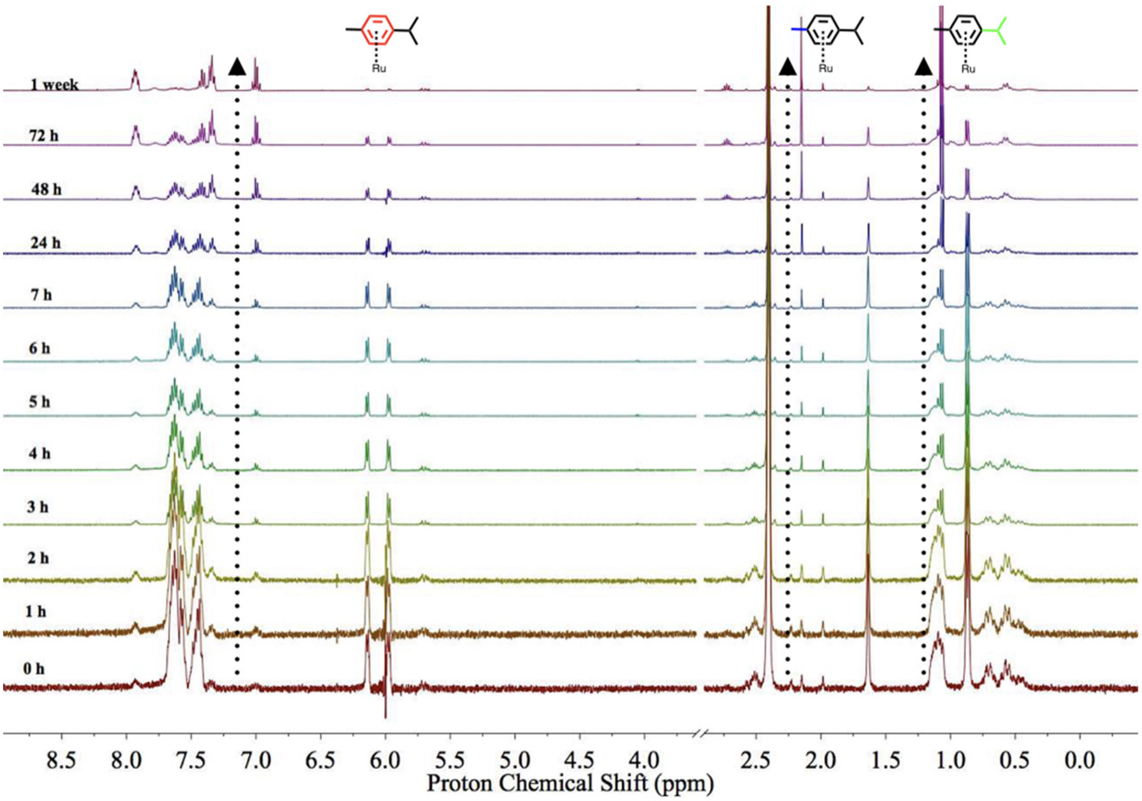

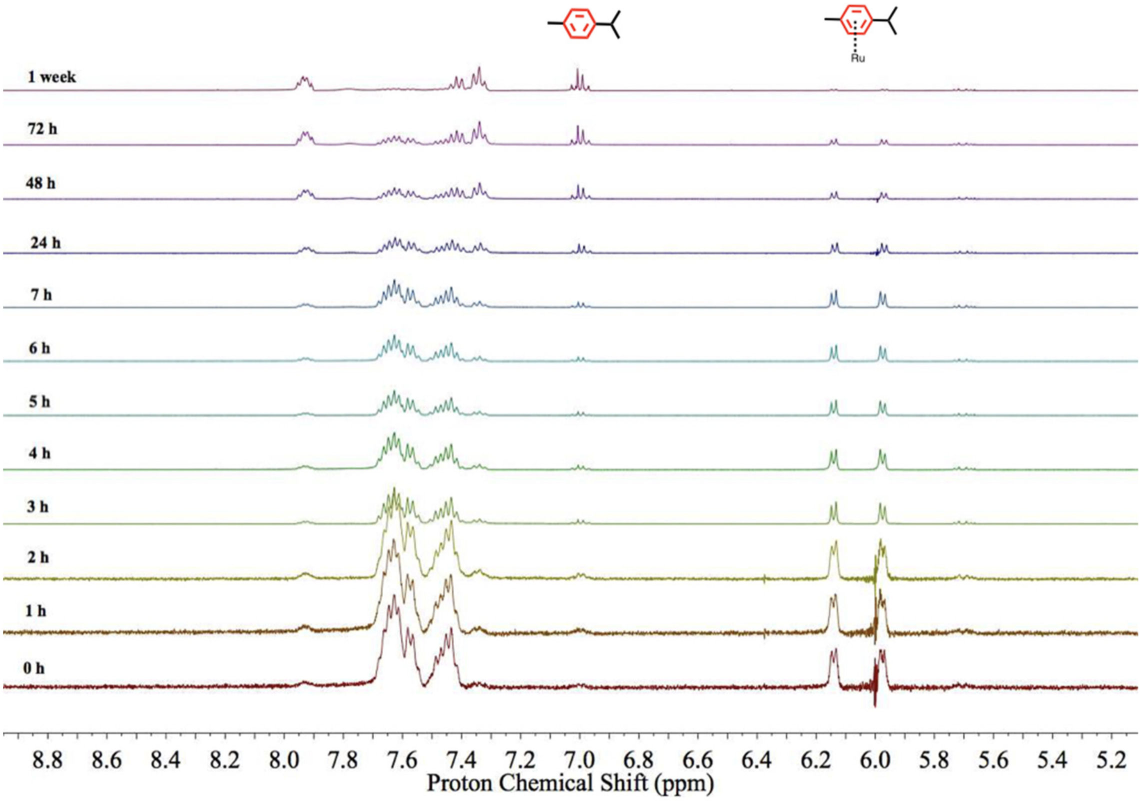

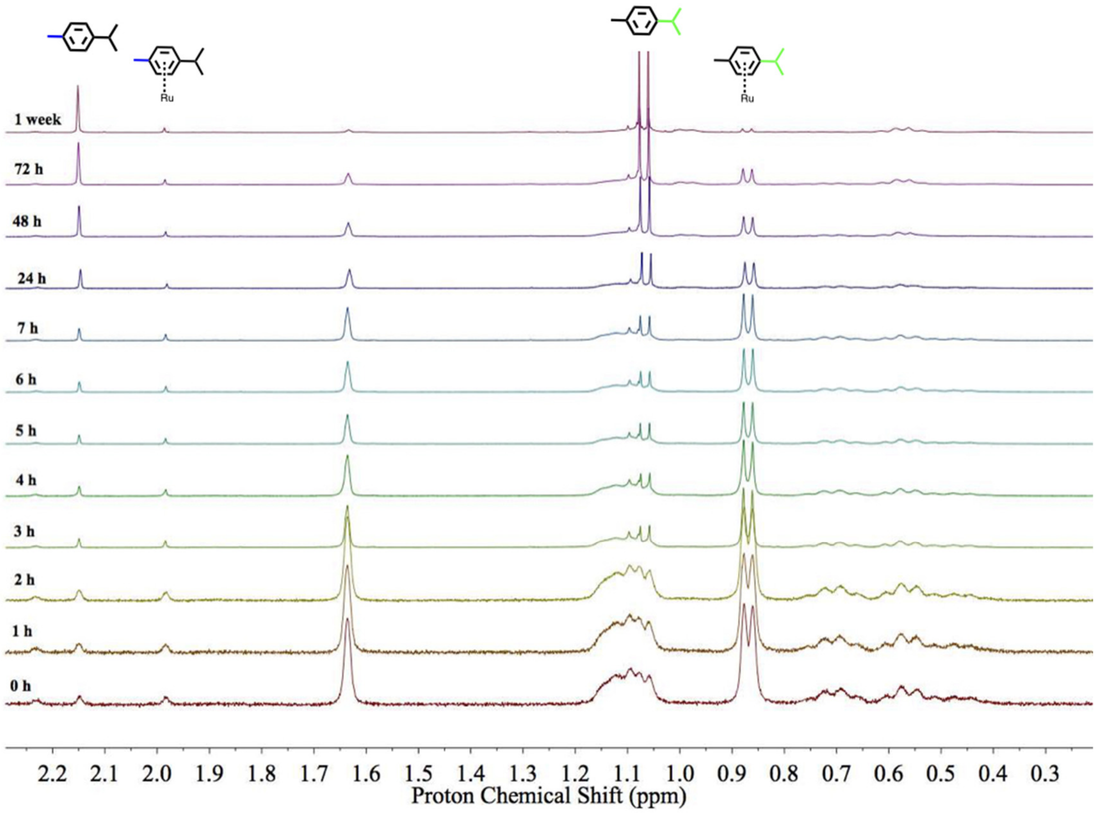

Figure S6. Selected ^{1}H spectra at different time of Complex *Ru3* ([RuCl(p-cym)({Ph_2P}$_2$N{C_6H_{11}})][BF_4]) in DMSO-d_6. Selected region for aromatic and aliphatic, respectively, at different time of complex Ru3.

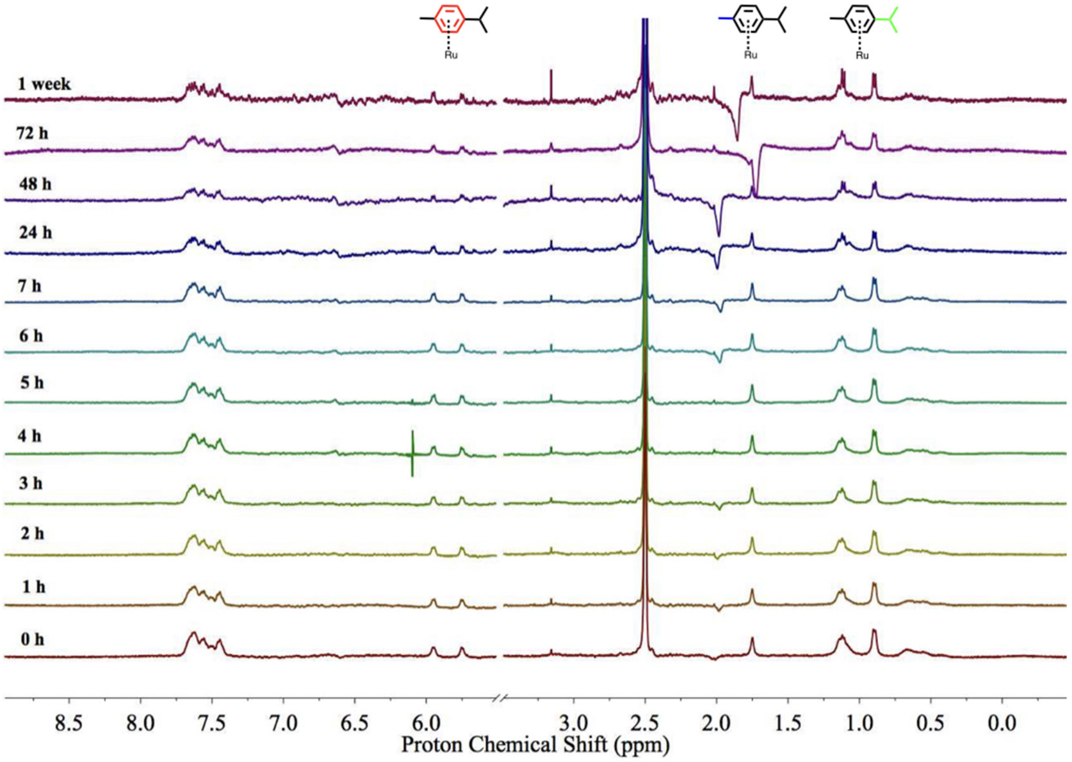

Figure S7. Selected ^{1}H spectra at different time of Complex *Ru3* ([RuCl(p-cym)({Ph_2P}$_2$N{C_6H_{11}})][BF_4]) in DMSO-d_6:D_2O / 1:3.

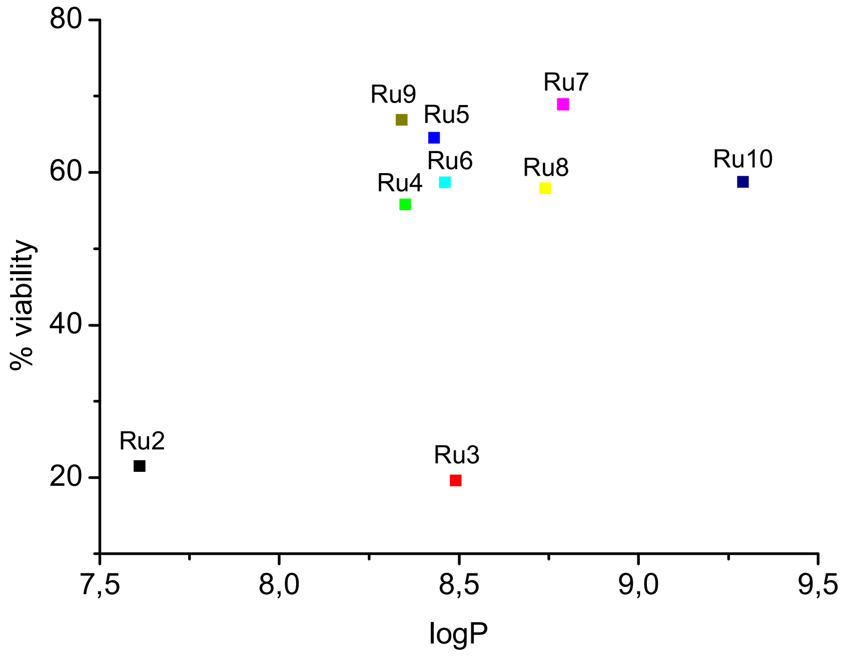

Figure S8. Graph of %viability obtained from the MTT experiment vs logP.

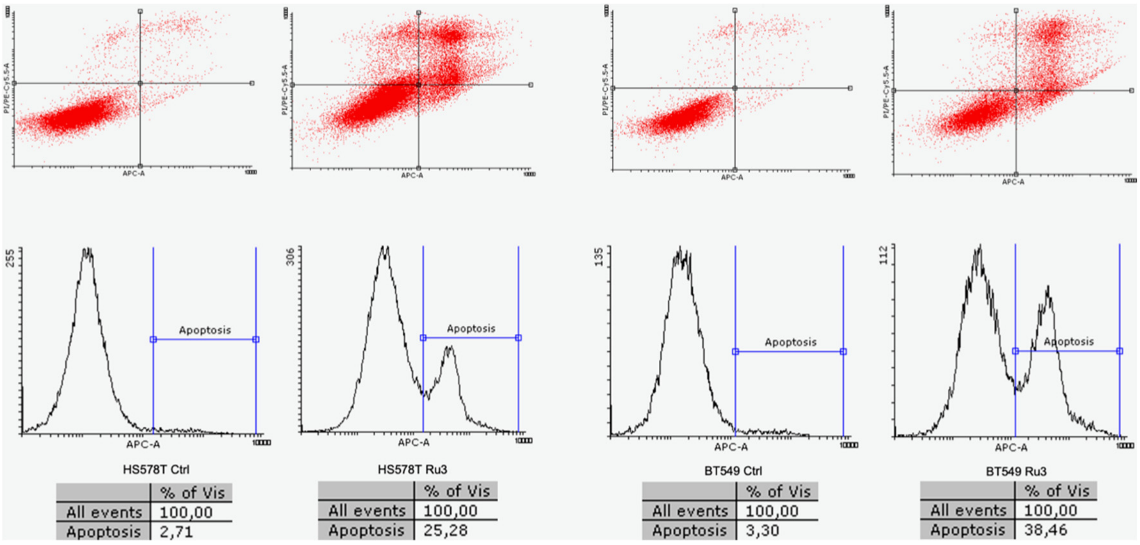

Figure S9. FAC Plot for HS578T and BT549.

Table S1. Principal bond distances and angles for compound *Ru3*.

Bond distances	Bond angles
Ru1 C3 2.240(16)	C3 Ru1 P2 159.3(5)
Ru1 C5 2.249(17)	C5 Ru1 P2 125.3(5)
Ru1 C1 2.261(14)	C1 Ru1 P2 101.3(4)
Ru1 C6 2.265(14)	C6 Ru1 P2 100.7(4)
Ru1 C2 2.273(15)	C2 Ru1 P2 124.7(4)
Ru1 P2 2.296(4)	P2 Ru1 C4 160.5(7)
Ru1 C4 2.299(12)	C3 Ru1 P1 130.2(5)
Ru1 P1 2.343(4)	C5 Ru1 P1 102.8(5)
Ru1 Cl1 2.383(3)	C1 Ru1 P1 155.8(5)
	C6 Ru1 P1 122.1(4)
	C2 Ru1 P1 164.4(4)
	P2 Ru1 P1 68.42(13)
	C4 Ru1 P1 105.3(5)
	P2 Ru1 Cl1 85.90(13)
	P1 Ru1 Cl1 87.71(14)

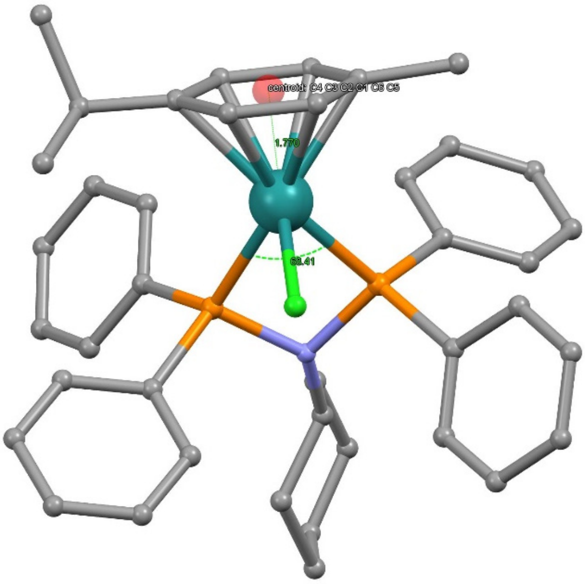

Table S2. Crystal data and structure refinement for compound Ru3.

**Compound**	**Ru3**
Formula	$C_{40}H_{45}BClF_4NP_2Ru$
M	825.04
CCDC	2076579
Crystal System	Monoclinic
Space group	$P2_1$
T [K]	300
a [Å]	10.3904(16)
b [Å]	18.4721(15)
c [Å]	10.7238(9)
β [deg]	104.915(4)
V [Å^3]	1988.9(3)
Z	2
Density [gcm^{-3}]	1.378
μ [mm^{-1}]	0.589
Observed reflections	19228
R_{int}	0.0964
R_1 [b] / wR^2 [c] [I>2σ(I)]	0.0489 / 0.1151
R_1 [b] / wR^2 [c] (all data)	0.0635 / 0.1279
GoF	1.132

[a] $S = [\sum w(F_0^2 - F_c^2)^2 / (N_{obs} - N_{param})]^{1/2}$ [b] $R_1 = \sum||F_0|-|F_c|| / \sum|F_0|$ [c] $wR_2 = [\sum w(F_0^2 - F_c^2)^2 / \sum wF_0^2]^{1/2}$ $w = 1/[\sigma^2(F_0^2) + (aP)^2 + bP]$ where $P = (\max(F_0^2,0) + 2F_c^2)/3$.

Table S3. Stability of *Ru1-Ru10* in DMSO-d_6 monitoring by ^{1}H-NMR.

**Complexes**	**50% degradation**	**Full degradation**
Ru1	6h	1 week
Ru2	24h	1 week
Ru3	7h	1 week
Ru4	7h	72h
Ru5	24h	1 week
Ru6	7h	72h
Ru7	5h	48h
Ru8	3h	48h
Ru9	-	-
Ru10	3-4h	48h

Table S4. Binding parameters (±2σ) obtained for the interaction of Ru3 and Ru8 with HSA compared to Ru0.

**COMP.**	**BIOMOL.**	**K_a x 10^{-4} (230nm)**	**K_a x 10^{-4} (278nm)**	**K_b x 10^{-4}**	**K_{sv} x 10^{-4}**	**n**
Ru0	HSA	16.2 ± 5.96	-	-	0.32 ± 0.10	1.11 ± 0.21
Ru3	HSA	3.37 ± 0.38	1.28 ± 0.43	4.02 ± 0.73	2.51 ± 0.26	1.05 ± 0.02
Ru8	HSA	4.25 ± 0.99	-	0.20 ± 0.002	2.04 ± 0.90	0.81 ± 0.07

Table S5. IC_{50} values for the most active compounds *Ru0*, *Ru2* and *Ru3*, and the ligands in every cell line used.

**Cell Line**	**IC_{50} (nM)**			
	**Ru0**	**Ru2**	**Ru3**	**L1-L9**
HACAT	-	-	350	>1000
MCF7	300	45	15	>1000
T47D	450	60	10	>1000
HS578T	-	-	250	>1000
BT549	-	-	45	>1000
SKBR3	700	80	60	>1000
BT474	800	180	120	>1000
OVCAR8	-	-	300	>1000
SKOV3	-	-	170	>1000
